# Absence of Association between Methylene Blue Reduced Susceptibility and Polymorphisms in 12 Genes Involved in Antimalarial Drug Resistance in African *Plasmodium falciparum*

**DOI:** 10.3390/ph14040351

**Published:** 2021-04-09

**Authors:** Mathieu Gendrot, Océane Delandre, Marie Gladys Robert, Francis Tsombeng Foguim, Nicolas Benoit, Rémy Amalvict, Isabelle Fonta, Joel Mosnier, Marylin Madamet, Bruno Pradines, on behalf of the French National Reference Centre for Imported Malaria Study Group

**Affiliations:** 1 Unité Parasitologie et Entomologie, Département Microbiologie et Maladies Infectieuses, Institut de Recherche Biomédicale des Armées, 13005 Marseille, France; ma.gendrot@laposte.net (M.G.); o.delandre@gmail.com (O.D.); m.gladys.robert@gmail.com (M.G.R.); francisfoguim@gmail.com (F.T.F.); nicobenoit73@hotmail.com (N.B.); remy_alt@yahoo.fr (R.A.); isabelle.fonta.09@gmail.com (I.F.); joelmosnier@orange.fr (J.M.); mmadamet@gmail.com (M.M.); 2 Aix Marseille Univ, IRD, SSA, AP-HM, VITROME, 13005 Marseille, France; 3 IHU Méditerranée Infection, 13005 Marseille, France; 4 Centre National de Référence du Paludisme, 13005 Marseille, France

**Keywords:** malaria, *Plasmodium falciparum*, antimalarial drug, methylene blue, resistance, in vitro, molecular marker

## Abstract

Half the human population is exposed to malaria. *Plasmodium falciparum* antimalarial drug resistance monitoring and development of new drugs are major issues related to the control of malaria. Methylene blue (MB), the oldest synthetic antimalarial, is again a promising drug after the break of its use as an antimalarial drug for more than 80 years and a potential partner for triple combination. Very few data are available on the involvement of polymorphisms on genes known to be associated with standard antimalarial drugs and parasite in vitro susceptibility to MB (cross-resistance). In this context, MB susceptibility was evaluated against 482 isolates of imported malaria from Africa by HRP2-based ELISA chemosusceptibility assay. A total of 12 genes involved in antimalarial drug resistance (*Pfcrt*, *Pfdhfr*, *Pfmdr1*, *Pfmdr5*, *Pfmdr6*, *PfK13*, *Pfubq*, *Pfcarl*, *Pfugt*, *Pfact*, *Pfcoronin*, and copy number of *Pfpm2*) were sequenced by Sanger method and quantitative PCR. On the *Pfmdr1* gene, the mutation 86Y combined with 184F led to more susceptible isolates to MB (8.0 nM vs. 11.6 nM, *p* = 0.03). Concerning *Pfmdr6*, the isolates bearing 12 Asn repetitions were more susceptible to MB (4.6 nM vs. 11.6 nM, *p* = 0.005). None of the polymorphisms previously described as involved in antimalarial drug resistance was shown to be associated with reduced susceptibility to MB. Some genes (particularly *PfK13*, *Pfugt*, *Pfact*, *Pfpm2*) did not present enough genetic variability to draw conclusions about their involvement in reduced susceptibility to MB. None of the polymorphisms analyzed by multiple correspondence analysis (MCA) had an impact on the MB susceptibility of the samples successfully included in the analysis. It seems that there is no in vitro cross-resistance between MB and commonly used antimalarial drugs.

## 1. Introduction

According to the World Health Organization (WHO) 2020 report [1], half of humanity is exposed to malaria in 87 countries in 2019 with 229 million malaria cases and 409,000 deaths (97% were African and 67% were children under five years old). *P. falciparum* parasites show resistance on different levels. First, parasites have developed an escape phenomenon involving the deletion of the *pfhrp2* (*P. falciparum* histidin-rich protein 2) gene that codes the main protein used in malaria rapid diagnostic tests for first line diagnosis in Africa [2]. Secondly, *P. falciparum* drug resistance to most antimalarial compounds has emerged in Southeast Asia and spread to Africa [3,4]. Resistance to artemisinin-based combination therapy (ACT), the last WHO recommended antimalarial drugs as first-line treatment for uncomplicated malaria, emerged in Western Cambodia, Myanmar, Thailand, and throughout Southeast Asia [5,6]. In this context, the development of new antimalarial drugs and new ACT partners became an emergency. In parallel, it is mandatory to identify mutated genes linked to resistance in order to avoid cross-resistance between drugs and choose good partners for new ACT therapies.

Methylene blue (MB), the oldest synthetic antimalarial, is again a promising drug after the break of its use as an antimalarial drug for more than 80 years and a potential partner for triple combination. MB showed potent in vitro activity at nanomolar range against *P. falciparum* and *P. vivax* clinical isolates [7,8,9,10,11,12,13,14,15]. Additionally, MB showed high synergistic effects in combination with dihydroartemisinin [16]. MB also showed a protective effect against a cerebral malaria in murine model infected with *P. berghei* [17,18,19]. MB is active against the gametocyte stages of *P. falciparum* and reduces the transmission of *P. yoelii* and *P. falciparum* [20,21,22,23,24,25,26]. Moreover, the mode of action of MB, targeting the *P. falciparum* glutathione reductase, differs from that of other antimalarial drugs [27]. MB also inhibits the polymerization of heme into hemozoine [28]. Moreover, the redox-cycling of MB affects homeostasis in the infected erythrocyte and the digestion of methemoglobin by *P. falciparum*, leading to inhibition of parasite growth [28]. Threshold value for in vitro reduced susceptibility to MB was estimated at 35 nM by statistical Bayesian analysis of the distribution of median effective concentration (EC_50_) estimates of 609 *P. falciparum* African isolates and 5.7% of the parasites displayed EC_50_ above this threshold [13]. Very few data are available on the involvement of polymorphisms on genes known to be associated with standard antimalarial drugs and parasite in vitro susceptibility to MB (cross-resistance).

The different genes and their polymorphisms associated with antimalarial drug reduced susceptibility, assessed in this work, were summarized in Table 1.

Numerous genes are believed to be involved in antimalarial drug reduced susceptibility and resistance, such as ATP-binding cassette (ABC) transmembrane transporter super family members. ABC transporters bind ATP in order to allow substrate to pass through the cell membrane against their solvent gradient in accordance with the cell needs. ABC transporter superstructure is mostly composed of transmembrane domains which comply in a canal shape [29,30]. ABC transporters are implicated in the draining of drugs out of the cytoplasm of cells. ABC transporters and their polymorphisms are known to be involved in drug resistance [31].

One of those is *Pfmdr1*, *Plasmodium falciparum* multi-drug resistance protein 1 (PF3D7_0523000), which codes for P-glycoprotein homologous (Pgh1), a protein homologous to efflux pumps of drugs involved in human anti-cancerous treatment [32]. Five single-nucleotide polymorphisms (SNP) were described, N86Y, Y184F, S1034C, N1042D and D1246Y [33]. *Pfmdr1* role was thought limited [34,35] but recent studies found that it might be involved in chloroquine, quinine, monodesethylamodiaquine, mefloquine, lumefantrine, and dihydroartemisinin susceptibility modulation [34,36,37].

Another ABC transporter gene is *Pfmdr5*, *P. falciparum* multi-drug resistance protein 5 (PF3D7_1339900). Repetitive amino acid motifs (DNNN and DHHNDHNNDNNN) in *Pfmdr5*, which codes a protein localized at the plasma membrane of the parasite [38], were associated with reduced in vitro susceptibility to lumefantrine or piperaquine [36,39]. Additionally, deletion of *Pfmdr5* in *P. falciparum* parasites resulted in a minor decrease of the inhibiting concentration 50% (IC_50_) to artemisinin but not in those to dihydroartemisinin, chloroquine, quinine, mefloquine, or lumefantrine [40].

Another ABC transporter gene involved in antimalarial drug resistance is *Pfmdr6*, *P. falciparum* multi-drug resistance protein 6 (PF3D7_1352100). The presence of 6 Asparagines (Asn) repeats was significantly associated with reduced susceptibility to lumefantrine [39], 7 Asn repeats with reduced susceptibility to lumefantrine or quinine [41,42], 8 Asn repeats with reduced susceptibility to piperaquine [39] and 9 Asn repeats with reduced susceptibility to dihydroartemisinin or quinine [41,42], according to several studies.

The gene *Pfcrt* (*P. falciparum* chloroquine resistance transporter) is well known now for its implication in chloroquine resistance. It belongs to the DMT (drug/metabolite transporters) superfamily. It is located on the digestive vacuole membrane of the parasite and increases the efflux of the chloroquine. Mutations in CRT, and more particularly, the 76T mutation, can cause a reduced intravacuolar concentration of chloroquine which can no longer inhibit hemozoin synthesis [43]. This mutation has been detected and validated by transfection in 2002 [44]. However, parasites with 76T mutation can be susceptible in vitro to chloroquine [45].

Those pumps present major implication in drug resistance in *P. falciparum* [46] but other proteins are involved in resistance.

In Southeast Asia, mutations in the propeller domain of the *PfK13* (*P. falciparum* Kelch 13) are linked to artemisinin resistance [47]. Until now 108 mutations have been found in Cambodia [48] and more particularly the mutations M476I, C580Y, R539T, Y493H, I543T and P574L are associated with artemisinin resistance in Asia [7]. However, the main K13 mutations involved in artemisinin resistance in Southeast Asia are not yet reported in Africa in the rare cases of clinical failures with ACT [49]. A new mutation (R561H), associated with in vitro resistance to artemisinin, has been recently identified in Rwanda [50,51]. Moreover, in the absence of mutation, a reduced *PfK13* transcriptional response led to longer parasite clearance time after artemether-lumefantrine treatment in African children [52].

*Pfpm2 (P. falciparum* Plasmepsin II) gene (PF3D7_1408000) codes a protease implicated in hemoglobin degradation. The augmentation of copy number of this gene is linked to piperaquine resistance in Cambodia [53,54].

*Pfdhfr* (*P. falciparum* dihydrofolate reductase) is a well-known gene, implicated in pyrimethamine *P. falciparum* resistance [55,56]. Mutations N51I, C59R and S108N are associated with in vitro resistance and clinical failures with pyrimethamine or proguanil [36].

*Pfubq* gene, *P. falciparum* HECT E3 ubiquitin ligase gene (PF3D7_0826100), codes one of the three enzymes interacting in the post-translational modifications of proteins by ubiquitin, known as ubiquitylation [57]. The ubiquitylation pathway could be a possible target for antimalarial drugs and might be involved in the resistance phenomenon [58]. Previous publications using the genome-wide association study (GWAS), showed mutations on the HECT E3 ubiquitin ligase gene in Senegalese isolates which were involved in pyrimethamine resistance [59]. Furthermore, HECT E3 ubiquitin ligase gene polymorphism might be involved in quinine and quinidine reduced susceptibility [60]. Another E3 ubiquitin-protein ligase (PF3D7_0627300), belonging to the RING-type E3 ligase family, showed association with chloroquine and amodiaquine susceptibility [61] but not with susceptibility to piperaquine [62].

The gene *Pfcoronin*, which codes the actin filament-organizing protein coronin, has been recently found to be associated with artemisinin resistance. Three mutations, G50E, R100K, and E107V, are identified in isolates from Senegal, that were adapted by artemisinin pressure in in vitro culture over four years [63,64,65]. The role of *Pfcoronin* mutations in in vitro artemisinin resistance was confirmed using CRISPR/Cas9 editing [66]. However, the three previously identified mutations in *Pfcoronin* were not detected in *P. falciparum* Senegalese isolates collected after clinical failures with ACT [67].

PfACT (*P. falciparum* Acetyl-CoA transporter) and PfUGT (*P. falciparum* UDP-galactose transporter) belong to the superfamily of major facilitator transporters and may be involved in intracellular transport of drugs in Plasmodium [68]. Some mutations in the two putative transporters have been associated with in vitro resistance to imidazolopiperazines [69].

Some mutations in the *Pfcarl* gene (*P. falciparum* cyclic amine resistance locus), which codes a protein involved in protein export, lead to in vitro resistance to imidazolopiperazines but not to artemisinin, chloroquine, or mefloquine [69,70,71,72,73]. Moreover, a new 734M mutation was found to be associated with reduced susceptibility to pyronaridine in *P. falciparum* African isolates [74].

Very few data are available on the involvement of polymorphisms on genes known to be associated with standard antimalarial drugs and parasite in vitro susceptibility to MB (cross-resistance). In this context, the MB susceptibility was evaluated against 482 isolates of imported malaria from Africa by HRP2-based ELISA chemosusceptibility assay. The 12 genes presented above were sequenced by Sanger method and quantitative PCR.

## 2. Results

A total of 482 samples were successfully evaluated in vitro for MB susceptibility. The origin of African isolates is unknown for four samples. The others came from Angola (2), Benin (13), Burkina Faso (27), Burundi (1), Cameroon (113), Central African Republic (38), Comoros (14), Democratic Republic of Congo (29), Djibouti (2), Gabon (31), Ghana (4), Guinea (17), Conakry Guinea (5), Equatorial Guinea (6), Madagascar (6), Mali (6), Morocco (1), Mozambique (1), Niger (4), Nigeria (5), Uganda (1), Ivory Coast (106), Rwanda (2), Senegal (9), Sierra-Leone (2), Sudan (1), Tanzania (1), Chad (10), and Togo (21).

Among the 482 African isolates included in the study, 32.7% of the parasites showed reduced susceptibility to chloroquine, 9.0% to desethylamodiaquine (the active metabolite of amodiaquine), 65.9% to mefloquine, 16.3% to dihydroartemisinin (the active metabolite of artemisinin derivatives), 19.3% to doxycycline, 4.7% to pyronaridine, 8.4% to artesunate and 0.5% of piperaquine.

The global mean IC_50_ (Inhibitory concentration 50%) to MB was 11.5 nM, with 5.2% (*n* = 25) of samples having an IC_50_ above the cut-off of reduced susceptibility of 35 nM. The mean IC_50_ in the reduced chemosusceptibility group was 48.4 nM.

### 2.1. Pfcrt

A total of 468 isolates were sequenced for *Pfcrt* gene displaying chemosusceptibility data. The mutated haplotype CVIET (positions 72 to 76) was present in 89 samples (19% of the 468 samples). The wild haplotype CVMNK was present in 346 isolates (74%). Thirty-three samples (7%) carried both wild and mutated haplotypes. The means MB IC_50_ for parasites harboring wild type haplotype (CVMNK), mutated haplotype (CVIET), and mixed haplotypes were 11.5 nM (95% Confident interval = 10.2–12.7 nM), 11.3 nM (95%CI = 8.9–13.8 nM), and 11.0 nM (95%CI = 6.9–15.0 nM), respectively. There is no significant difference between the three groups in MB susceptibility (Welch’s *t*-test, *p* > 0.8).

The 356T mutation was present in 9.2% of the samples. The means MB IC_50_ for parasites wild type (I356) and mutated parasites (356T) were 12.3 nM (95%CI = 10.3–13.6 nM) and 13.6 nM (95%CI = 8.7–18.5 nM), respectively. There is no significant difference between the 2 groups in MB susceptibility (Welch’s *t*-test, *p* = 0.61).

### 2.2. Pfdhfr

A total of 470 isolates were successfully sequenced for *Pfdhfr* gene. The mutation 108N was present in 430 samples (91.5%). The mutations 51I and 59R were present in 406 (86.4%) and 426 isolates (90.6%), respectively. The mutated haplotype IRN was present in 406 isolates (86.4%). The means MB IC_50_ for parasites harboring wild type haplotype (NCS) and mutated haplotype (IRN) were 11.6 nM (95%CI = 10.4–10.9 nM) and 11.3 nM (95%CI = 7.1–15.4 nM), respectively. There is no significant difference between the two groups in MB susceptibility (Welch’s *t*-test, *p* = 0.86).

### 2.3. Pfmdr1

A total of 480 isolates were successfully sequenced for *Pfmdr1* gene and the results are presented in Table 2. Thirty-seven isolates harbored the 86Y mutation (8.5%), 433 samples (90.2%) were wild type N86 and 10 isolates (1.3%) were mixed. On position 184, 237 isolates (53.6%) were muted and 29 isolates (6.2) had mixed population. All the samples were wild type for S1034 and N1042 and only two isolates (0.5%) were muted on position 1246Y. There is no significant difference susceptibility to MB between the different haplotypes. Only parasites bearing the 86Y-184F haplotypes are more susceptible to MB than the other ones (8.0 nM vs. 11.6 nM; *p* = 0.03).

### 2.4. Pfmdr5

We successfully sequenced 379 isolates. For simplification, the motifs DNNN and DHHNDHNNDNNN were renamed R1 and R2. The results are presented in Table 3. There is no significant difference in susceptibility to MB between the different genotypes of *Pfmdr5*. However, parasites with less than nine DNNN motifs seem to be less susceptible to MB than isolates with at least nine DNNN motifs. However, this difference was not significant (*p* = 0.08).

Contrary to previous works published, the sequencing of our imported malaria isolates gave longer DNA fragment on chromatogram data. A new polymorphism was identified, in amino acid position 686 to 694 (p.686_694 N). This region is a repetition of nine successive Asparagine (Asn). Sequencing revealed a variation number of Asn, from 5 to 14. Although there were 349 isolates successfully sequenced and dispatched in 10 groups (Table 4). There is no significant difference susceptibility to MB between the different genotypes according to the number of 686_694 Asn repeats in *Pfmdr5*.

### 2.5. Pfmdr6

Concerning the *Pfmdr6* gene, 482 isolates were successfully sequenced. The results are shown in Table 5. Eleven polymorphisms were identified, ranging from 4 to 18 Asn repeats. The highest proportion of isolates in our study had six Asn repeats (45% of the samples). The *P. falciparum* parasites with 12 Asn repeats were more susceptible to MB (4.6 nM vs. 11.6 nM; *p* = 0.005). However, the 12 Asn group included only seven isolates.

### 2.6. PfK13

A total of 475 isolates were successfully sequenced for *Pfk13* gene. All the *P. falciparum* isolates were wild type.

### 2.7. Pfubq

A total of 214 isolates were successfully sequenced for *Pfubq* gene. Only isolates collected in 2015–2016 were sequenced. The 113N mutation was present in 29.9% of the samples. The means MB IC_50_ for parasites wild type (D113) and mutated parasites (113N) were 10.2 nM (95%CI = 8.5–11.8 nM) and 9.2 nM (95%CI = 6.8–11.7 nM), respectively. There is no significant difference between the two groups in MB susceptibility (Welch’s *t*-test, *p* = 0.55).

### 2.8. Pfcarl

A total of 268 isolates, collected between 2015 and 2016, were successfully sequenced for *Pfcarl* gene. The 784N mutation was present in only one sample and the 734M in 20 isolates (7.5%). The means MB IC_50_ for parasites wild type (K734) and mutated parasites (734M) were 11.9 nM (95%CI = 10.0–13.9 nM) and 8.7 nM (95%CI = 1.5–15.5 nM), respectively. There is no significant difference between the two groups in MB susceptibility (Welch’s *t*-test, *p* = 0.09).

### 2.9. Pfugt

All the 259 isolates collected between 2015 and 2016 and successfully sequenced were wild type for *Pfugt* gene.

### 2.10. Pfact

All the 259 *P. falciparum* samples collected between 2015 and 2016 and successfully sequenced were wild type for *Pfact* gene.

### 2.11. Pfcoronin

A total of 310 isolates, collected between 2018 and 2019, were successfully sequenced for *Pfcoronin* gene. Only one mutation was detected on position 76 (76S) with a prevalence of 10.3% (*n* = 32). The means MB IC_50_ for parasites wild type (P76) and mutated parasites (76S) were 12.6 nM (95%CI = 11.2–14 nM) and 9.8 nM (95%CI = 5.6–13.9 nM), respectively. There is no significant difference between the two groups in MB susceptibility (Welch’s *t*-test, *p* = 0.11).

### 2.12. Pfpm2

All the 455 *P. falciparum* isolated successfully quantified harbored only one copy of *Pfpm2* gene.

### 2.13. Multiple Correspondence Analysis (MCA)

Since R code can only support data with full information on each row (meaning that a missing value for a genotype exclude the isolate) and requires components with at least two variables, we had to exclude data on *Pfubq*, *Pfcarl*, and *Pfcoronin* due to a lower number of samples and *PfK13*, *Pfugt*, *Pfact*, and *Pfmp2* due to an absence of genetic variability on the polymorphisms analyzed. The analysis was performed only for *Pfmdr5*, *Pfmdr6*, *Pfmdr1*, *Pfcrt*, and *Pfdhfr*. A total of 271 isolates were successfully included.

**Figure 1:**
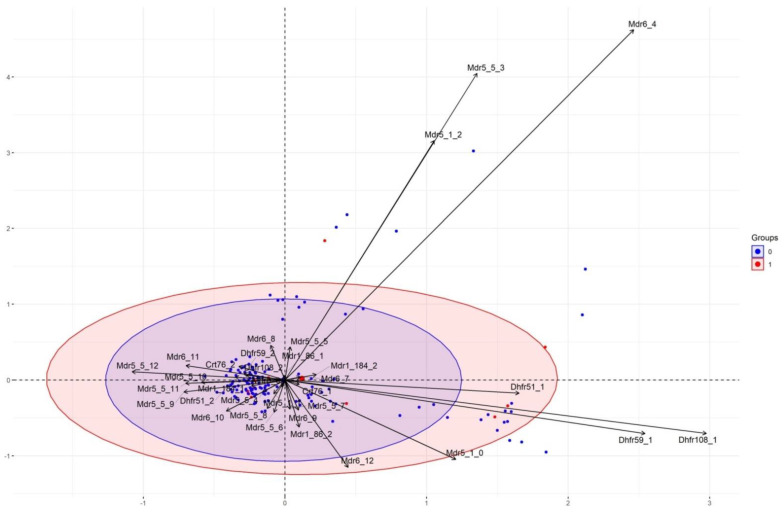
Multiple correspondence analysis biplot of *Pfmdr5*, *Pfmdr6*, *Pfmdr1*, *Pfcrt*, and *Pfdhfr* gene polymorphisms in 271 African *P. falciparum* isolates (in blue parasites with IC_50_ < 35 nM and in red parasites with IC_50_ > 35 nM; Dhfr51_1 represents the haplotype N51 on *Pfdhfr*, Dhfr51_2 for 51I, Dhfr59_1 for C59, Dhfr59_R for 59R, Dhfr108_1 for S108, Dhfr108_2 for 108N, Mdr1_86_1 for N86, Mdr1_86_2 for 86Y, Mdr1_184_1 for Y184, Mdr1_184_2 for 184F, Crt76_1 for CVMNK, Crt76_2 for CVIET, Mdr5_x_y, x as pattern R1 and y as pattern R2, Mdr6_z, z as Asn repeat number).

No correlation was observed on the biplot between principal components (PC) due to the superposition of the two ellipses representing the two groups (IC_50_ below or above the cut-off of 35 nM for reduced susceptibility to MB) and an absence of a cluster outside the ellipses (Figure 1).

## 3. Discussion

Different gene coding pumps, that are involved in antimalarial drug trafficking and resistance, were assessed in this study. Some of these transporters are implicated in the transport of quinolines, which accumulate in the vacuole of *P. falciparum* parasites, bind to hematin and inhibit the polymerization of heme into hemozoin [75]. The *Pfcrt* gene is involved in chloroquine and amodiaquine resistance [76,77]. In the present work, 32.7% of the parasites showed reduced susceptibility to chloroquine while 26% of the isolates harbored the mutated haplotype CVIET (19% with unique haplotype CVIET and 7% with mixed haplotypes CVIET and CVMNK). The 75E and 76T mutations showed a positive predictive value for in vitro resistance to chloroquine of 51.6 and 60.0%, respectively [45]. Parasites with a 76T mutation can be susceptible in vitro to chloroquine [45]. However, we detected in the present study a higher prevalence of parasites with reduced in vitro susceptibility to chloroquine than parasites harboring mutated haplotype CVIET, suggesting that additional factors may be involved in chloroquine resistance. The MB in vitro susceptibility is not modulated by the polymorphisms associated with chloroquine and amodiaquine, suggesting no cross-resistance between MB and the two quinolines.

The *Pfmdr1* N86Y mutation is involved in parasite susceptibility to most of the partner drugs of ACT, including artemisinin, lumefantrine, amodiaquine, mefloquine, and piperaquine [37,78]. Moreover, clinical assays in East Africa have shown the selection of the N86 allele in recurrent infections after treatment with artemether plus lumefantrine [79,80] or artesunate plus mefloquine [81]. The presence of the N86 mutation was a risk factor for recrudescence in patients who were treated with artemether-lumefantrine [82]. In Uganda, the use of dihydroartemisinin-piperaquine selected for the 86Y mutation [83,84]. Moreover, the 86Y mutation was found to be associated with treatment failures after amodiaquine monotherapy [85,86,87] or artesunate-amodiaquine [82,88]. Additionally, Veiga and colleagues showed that transfected parasites with the N86-184F and N86-Y184 haplotypes were less susceptible to lumefantrine than parasites bearing the 86Y-184F or 86Y-Y184 haplotypes [78]. In this study, the 86Y-184F haplotype was more susceptible than the N86-Y184, N86-184F, or 86Y-Y184 haplotypes. However, this haplotype represents only 5.3% of all the parasites.

Repetitive amino acid motifs (DNNN—DHHNDHNNDNNN) in *Pfmdr5* are associated with reduced in vitro susceptibility to lumefantrine in *P. falciparum* isolates from around the world [39], or piperaquine in Senegalese parasites [36]. None of the repetitive motifs are associated with MB susceptibility. However, parasites with less than nine DNNN motifs seem to be less susceptible to MB than isolates with at least nine DNNN motifs. However, this difference was not significant (*p* = 0.08). In a previous study, the Senegalese parasites with eight or more copy repeats of DNNN in *pfmdr5* were significantly more susceptible to piperaquine [36]. Parasites with at least nine DNNN motif repeats seem to be less susceptible to both MB and piperaquine. Moreover, a new polymorphic region with the repetition of Asn residues between the amino acid positions 686 to 694 was detected. However, MB susceptibility is not modulated by the number of Asn repeats.

The presence of six Asn repeats in the polymorphic microsatellite region of *Pfmdr6* (amino acid positions 103 to 109 in 3D7) was significantly associated with reduced susceptibility to lumefantrine [39], seven Asn repeats with reduced susceptibility to lumefantrine or quinine [41,42], eight Asn repeats with reduced susceptibility to piperaquine [39], and nine Asn repeats with reduced susceptibility to dihydroartemisinin or quinine [41,42], according to several studies. However, from one study to another, the results are not consistent. In this study, the parasites with 12 Asn repeats were more susceptible to MB than the others. However, these parasites are very few (1.4%).

All the *P. falciparum* parasites were wild type for *Pfact* and *Pfugt* genes. These two genes did not present enough genetic variability to conclude on the absence of association between polymorphisms and MB susceptibility.

All the other genes coding non-transporter proteins involved in antimalarial drug resistance, like *PfkK13* associated with artemisinin resistance [6,47,48], *Pfcoronin* associated with artemisinin resistance [63,66], *Pfpm2* associated with piperaquine resistance [53,54], *Pfdhfr* associated with pyrimethamine resistance [89], *Pfcarl* associated with imidazolopiperazine resistance and potential reduced susceptibility to artemisinin, chloroquine or mefloquine [70,71,72], and *Pfubq* associated with chloroquine, amodiaquine or piperaquine [61,62], seem to not be associated with reduced susceptibility to MB. For *PfK13* and *Pfpm2*, it is difficult to conclude that there is an absence of association between the polymorphisms analyzed and MB susceptibility due to the absence of genetic variability.

According to the statistical analysis performed using R, we showed that none of the polymorphisms of *Pfmdr5*, *Pfmdr6*, *Pfmdr1*, *Pfcrt*, and *Pfdhfr* had an impact on the MB susceptibility of the samples successfully included in the analysis.

## 4. Materials and Methods

### 4.1. Sample Collection

A total of 482 venous blood samples, collected between 2015 to 2019 from patients hospitalized in France with imported malaria from an African malaria-endemic country, was sent within 72 h after collection from different civilian or military hospitals of the French National Reference Centre for Imported Malaria network to the French National Reference Centre for Malaria (IRBA, IHU Méditerranée Infection Marseille, Marseille, France). Epidemiological and clinical data were collected for each isolate. Species identification was determined on blood smear and confirmed by real-time PCR [90].

### 4.2. Ex Vivo Assay

MB, methylthioninium chloride Proveblue^®^, was provided by Provepharm SAS (Marseille, France). After dissolution, 14 concentrations of MB ranging from 0.1 nM to 500 nM were prepared in water.

The susceptibility of the isolates to MB was assessed ex vivo by ELISA HRP2 assay without culture adaptation. The in vitro test consisted of 100 µL of parasitized erythrocytes (final parasitemia at 0.5% and final hematocrit of 1.5%) aliquoted into 96-well plates. Each plate is pre-dosed with a concentration gradient of MB. The plates were incubated at 37 °C for 72 h under controlled atmosphere of 85% N_2_, 10% O_2_, and 5% CO_2_. Plates were frozen for 24 h at −20 °C, then thawed one hour at 37 °C.

The HRP2 ELISA-based assay Malaria Ag Celisa kit (ref KM2159, Cellabs PTY LDT, Brookvale, Australia) was used to estimate the parasite growth [91]. The inhibiting concentration 50% (IC_50_) was estimated by non-linear regression (ICEstimator version 1.2).

A chloroquine-resistant strain W2 (Indochina origin, obtained from MR4, Charlottesville, VA, USA), genetically controlled every two weeks, was used to each batch of plates used between 2013 and 2019 in three to six independent experiments [92,93].

### 4.3. Gene Sequencing

*Pfcrt* [94], *Pfdhfr* [92], *Pfmdr1* [37], *Pfmdr5* [36], *Pfmdr6* [42], *PfK13* [95], *Pfubq* [91], *Pfcarl* [74], *Pfugt* [74], *Pfact* [74], and *Pfcoronin* [66] were sequenced by Sanger method as previously described. The copy number of *Pfpm2* was quantified by quantitative PCR as previously described [96,97].

Sequencing was performed on ABI Prism 3100 analyzer (Applied Biosystems, Foster City, CA, USA). The Vector NTI advance^TM^ software (version 11, Invitrogen, Cergy Pontoise, France) and Codon Code aligner (Codon Code corporation, Centerville, OH, USA) were used to analysis chromatograms.

### 4.4. Statistical Analysis

R software with ggplot2 package for Dot plots, Inserm UPMC biostaTGV (http://websenti.u707.jussieu.fr/ (accessed on 26 March 2021)) tool and Excel statistical resource pack Real statistics ANOVA (Charles Zaiontz—http://realstatistics.com (accessed on 26 March 2021)) were used in order to perform all statistical tests of this project.

The Multiple correspondence analysis (MCA) was performed using R package *FactoMineR* [98] and *Factoextra* (http://www.sthda.com/english/rpkgs/factoextra (accessed on 26 March 2021)) on RStudio version 1.3.1093. The objective of MCA is to identify direction (the principal components) for which the variation of the data is maximal, and thus those directions which are linked to a genotype in particular can explain the behavior of the phenotype of our isolates.

## 5. Conclusions

The modest association for *Pfmdr1*, *Pfmdr5*, and *Pfmdr6* limited the interpretation of the present results. It is imperative to add more isolates from other areas of the world, particularly isolates which present a greater genetic variability for the genes analyzed. It would be necessary to evaluate additional genes like amplification of the *Pfmdr1* gene that confers reduced susceptibility to mefloquine [81] or increased susceptibility to piperaquine [99], polymorphisms of *Pfmrp1* and *Pfmrp2* (*P. falciparum* multidrug-resistance associated proteins) which mediate the efflux of glutathione, chloroquine, quinine or piperaquine [100], or amplification of the *Pfpm3/1* gene (*P. falciparum* Plasmepsin III) which is associated with piperaquine reduced susceptibility [101]. The absence of association between in vitro susceptibility to MB and polymorphisms of the common genes involved in antimalarial drug resistance suggest an absence of cross-resistance between MB and standard antimalarial drugs. MB is a potential partner for triple combination. The absence of association with the genes potentially involved in reduced susceptibility to lumefantrine (*Pfmdr1*, *Pfmdr5*, *Pfmdr6*), to amodiaquine (*Pfcrt*, *Pfmdr1*, *Pfubq*), to piperaquine (*Pfmdr5*, *Pfmdr6*, absence of amplification of *Pfmp2*) and to artemisinin (*Pfcoronin* and absence of polymorphisms on *PfK13*), suggests that MB could be combined with artemisinin derivative and amodiaquine, lumefantrine, or piperaquine. Moreover, previous data showed that MB improved the in vitro antiplasmodial activity of amodiaquine and dihydroartemisinin [16]. The combination MB-artesunate-amodiaquine showed high schizonticidal activity and efficacy against malaria [102,103].

## Figures and Tables

**Table 1:** Genes and their polymorphisms involved in modulation of the in vitro susceptibility to antimalarial drugs, assessed in the present work.

Gene	Polymorphisms Previously Described	Association with Antimalarial Susceptibility Modulation	References
*Pfmdr1*	N86Y, Y184, S1034C, N1042D, D1246Y mutations	chloroquine, quinine, monodesethylamodiaquine, mefloquine, lumefantrine, dihydroartemisinin	[33,34,36,37]
*Pfmdr5*	DNNN, DHHNDHNNDNNN repetitions	lumefantrine, piperaquine	[36,39]
*Pfmdr6*	Asn repetitions between amino acid positions 103 to 109 in 3D7	lumefantrine (6 and 7 Asn), piperaquine (8 Asn), dihydroartemisinin (9 Asn)	[39,41,42]
*Pfcrt*	M74I, N75E, K76T	chloroquine, amodiaquine	[44]
*PfK13*	M476I, Y493H, R539T, I543T, R561H, P574L, C580Y mutations	artemisinin	[7,47,48,50,51]
*Pfmp2*	Gene copy amplification	piperaquine	[53,54]
*Pfdhfr*	N51I, C59R, S108N mutations	pyrimethamine, proguanil	[36,55,56]
*Pfubq*	D113N mutation	chloroquine, amodiaquine	[61]
*Pfcoronin*	G50E, R100K, E107V	artemisinin	[63,66]
*Pfact*	A94T, R108K, S110R, D165N, G559K mutations	imidazolopiperazines	[69]
*Pfugt*	F37V	imidazolopiperazines	[69]
*Pfcarl*	K734M, L830V, S1076N/I, V1103L, I1139K	pyronaridine, imidazolopiperazines	[69,70,71,72,74]

**Table 2:** Association between the *Pfmdr1* haplotypes analyzed among 480 clinical *P. falciparum* African isolates and ex vivo susceptibility to methylene blue.

Haplotype	Number	Frequency (%)	Mean IC_50_ in nM	95% CI in nM	*p*-Value
N86 vs. 86Y	433/37	90.2/8.5	11.7/9.7	10.5–12.8/5.9–13.5	0.31
Y184 vs. 184 F	209/237	44.0/49.8	11.4/11.1	9.8–13.0/9.6–12.5	0.78
D1246 vs. 1246 Y	441/2	99.5/0.5	11.3/7.4		
N86-Y184 vs. N86-184F	187/209	47.2/52.8	11.2/11.6	9.5–12.9/10.0–13.1	0.74
N86-184F vs. all	209/260	44.6/55.4	11.6/11.4	10.0–13.1/10.0–12.7	0.58
86Y-184F vs. all	23/446	4.9/95.1	8.0/11.6	3.3–12.7/10.6–12.8	0.03
86Y-Y184 vs. all	13/456	2.8/97.2	11.4/12.9	10.4–12.5/6.6–19.3	0.70

**Table 3:** Association between the *Pfmdr5* genotypes analyzed among 379 clinical *P. falciparum* African isolates and ex vivo susceptibility to methylene blue.

Allele	Number	Frequency (%)	Methylene Blue IC_50_ in nM		
			Mean	95% CI	*p*-Value
3R1-1R2	3	0.8	10.3		
4R1-1R2	3	0.8	9.3		
5R1-1R2	110	29.0	12.7	10.3–15.1	0.76 ^a^
6R1-1R2	82	21.6	13.3	10.5–16.1	0.46 ^a^
7R1-1R2	101	26.6	12.4	9.6–14.9	0.99 ^a^
8R1-1R2	22	5.8	10.7	5.3–16.1	0.47 ^a^
9R1-1R2	7	1.8	7.4		
10R1-1R2	3	0.8	8.5		
11R1-1R2	1	0.3	8.9		
12R1-1R2	1	0.3	0.2		
13R1-1R2	1	0.3	17.5		
5R1-2R2	13	3.4	11.7	4.7–13.4	0.83 ^a^
0R2	4	1.1	9.1		
1R2	351	92.6	12.1	10.8–13.4	0.36 ^a^
2R2	24	6.3	13.3	8.2–18.3	0.67 ^a^
≤8R1-1R1	349	84.6	12.6	11.2–13.9	0.08 ^b^
>8R1-1R1	13	3.2	8.0	1.0–14.9	0.08 ^b^

^a^ Statistical analysis used Welsh two-sample *t* test to compare IC_50_ for parasites bearing these genotypes versus those that do not have. ^b^ Statistical analysis used Welsh two-sample *t* test to compare IC_50_ for parasites bearing ≤8R1-1R1 versus those that have >8R1-1R1.

**Table 4:** Association between the *Pfmdr5* 686_694 Asn repeats among 349 clinical *P. falciparum* African isolates and ex vivo susceptibility to methylene blue.

Asn Repetition	Number	Frequency (%)	Mean IC_50_ in nM	95% CI in nM	*p*-Value
5	2	0.5	11.9		
6	6	1.7	15.7	5.8–25.6	0.53
7	28	8.0	11.2	6.6–15.8	0.72
8	56	16.0	13.2	10.0–16.5	0.47
9	177	50.7	12.7	10.9–14.5	0.31
10	51	17.8	10.3	5.1–15.5	0.45
11	23	6.5	10.3	5.1–15.5	0.45
12	2	0.5	8.9		
13	2	0.5	3.4		
14	3	0.8	12.0		

Statistical analysis used Welsh two-sample *t* test to compare IC_50_ for parasites bearing these genotypes versus those that do not have.

**Table 5:** Association between *Pfmdr6* Asn repeats among 482 clinical *P. falciparum* African isolates and ex vivo susceptibility to methylene blue.

Asn Repetition	Number	Frequency (%)	Mean IC_50_ in nM	95% CI in nM	*p*-Value
4	5	1	13.3	2.8–23.7	0.81
5	1	0.2			
6	217	45.0	11.6	10.0–13.2	0.98
7	71	14.7	10.6	7.8–13.3	0.42
8	83	17.2	12.5	9.9–15.0	0.41
9	64	13.3	12.8	9.9–15.8	0.46
10	25	5.2	10.6	6.0–15.3	0.62
11	7	1.5	11.4	2.6–20.2	0.95
12	7	1.5	4.6	0.1–13.4	0.01
14	1	0.2			
18	1	0.2			

Statistical analysis used Welsh two-sample *t* test to compare IC_50_ for parasites bearing these genotypes versus those that do not have.

## Data Availability

The data presented in this study are available on request from the corresponding author.
